# Exploring VR in municipal mental health services: A pilot study

**DOI:** 10.1177/20556683251336126

**Published:** 2025-04-28

**Authors:** Solveig Osborg Ose, Hedvig Amanda Lestander, Per Lund Hoffmann, Yvonne Bokseth, Lotte Sundnes, Nina Onsaker Skjelbred, Ingvild Halset Bævre, Kristin Thaulow

**Affiliations:** 1 Health Services Research, 555969 SINTEF, Trondheim, Norway; 2 25574 Trondheim Municipality, EPHOR, Norway

**Keywords:** virtual reality (VR), mental health services, exploration, training-programme, piloting

## Abstract

**Introduction:** The study reports the results from piloting an 8-step program for social skills training using VR technology in a municipal mental health service.

**Method:** 14 mental health service users were recruited to test the 8-step programme. Eight mental health professionals delivered the programme and registered information about the participants and their experiences.

**Results:** All participants lived socially isolated lives before they started, and various social and personal problems contributed to the complexity of their situation. Seven participants completed the programme, while seven did not. Two participants did not complete because they stopped isolating themselves before completing and these two, and the seven that completed the programme, experienced a positive effect on their social skills. Two of those who did not complete were able to attend group therapy sessions instead, and two were too ill to complete. The last one just stopped showing up to the appointments.

**Conclusion:** The pilot study shows promising results and efforts should now be made to develop relevant, flexible, and high-quality VR scenarios, as this technology has the potential to help socially isolated persons become more socially active and thus increase their quality of life.

## Introduction

Twenty years ago, it was established that VR-based mental health therapy was more effective than no treatment.^1^ Today, we have more evidence of the effect of VR exposure therapy in anxiety disorders and depression^2,3^ and post-traumatic stress disorders^4^ and increasing evidence that focusing on reducing the response to triggers or “cues” that trigger unwanted behaviours such as substance abuse can be practised in VR.^5^ However, treatments based exclusively on virtual exposure to drug-related cues have shown heterogeneous results.^6^

VR has also been used in recreation programs to enhance the lives of long-term care residents^7,8^ and during the different phases of cancer treatment to reduce distress.^9^

The evidence of a positive effect of social skills training using VR for individuals with autism spectrum disorder is increasing,^10–12^ and VR is found to potentially be effective for training social skills in people with schizophrenia.^13–15^

In specialist mental health treatment, advanced computer-generated scenarios in VR are found to provide immersive, controlled environments with high ecological validity and personalisation potential.^16^ However, to our knowledge VR has not been explored in primary mental health services.

Implementation of digital health technologies into clinical practice has been challenging, and lack of knowledge and engagement from the mental health care workforce have been suggested as one of the barriers to implementation.^17^ Patients and clinicians have also described several barriers to the successful implementation of VR technology, including discomfort, boredom, lack of immersion and difficulties using the technology, unrealistic scenarios, time processing capability of the system, and user acceptance.^18,19^

Collaboration between mental health professionals and VR technology developers has been suggested to facilitate the integration of technological innovations into existing mental health services.^20^ However, we suggest that exploration and testing of VR in mental health services could be done with support from health services researchers and that a systematic approach, where small steps are taken over a longer period, might increase the probability of implementation in the long run.

Mental health professionals in public services would want to be involved in exploring technology if they believe the technology increases the quality of the services, that is — the service users receive better help and support than without the technology.

In this study, we describe how mental health professionals and mental health service users experienced the use of VR through piloting a social skills training programme.

## Methods

### Setting

Norway is a high-income country located in Scandinavia in Northern Europe. Mental health services in the country operate at the municipal level (i.e., primary health care) and the specialist level. Municipal-level responsibilities include prevention of health and social problems, assessment of functional abilities, early intervention and rehabilitation, follow-up, psychosocial support and counselling, and referral to specialist health services.

The municipality where this study was conducted is the third-largest municipality in Norway and provides a broad spectrum of services for those with mental health problem (e.g., housing services, mental health services for older adults, family support, and various therapeutic services).

### Exploring VR and the project establishment

The project started in 2018 with an all-day workshop with mental health professionals and health services researchers, where the potential for the use of VR in the treatment of severe mental health illness was explored. We concluded that one of the uses of VR technology with the greatest potential was helping individuals who had isolated themselves and needed training in social skills and everyday activity to enable them to have more active social lives. We established a research project and obtained funding to develop and pilot the VR programme.^21^ The internal project group in the municipal mental health service was established at the same time.

### Ethics

The research project was approved by the regional Research Ethics Committee in mid-Norway in January 2020 (REK 66949/2020). The researchers interacted freely with the mental health services employees and the psychologist who developed the prototype, but not with the three codeveloping service users nor the service users who tested the programme, i.e., the service users remained anonymous to the researchers. All participants provided written consent to participate in the study.

### Development of a social skills training programme using VR technology

To develop the social skills training programme, one mental health service user worked closely with the psychologists and mental health professionals to develop the prototype of the programme during 25 VR-based training sessions that combined psychoeducation and social skills training. After drafting the first programme version, two additional service users were recruited to serve as codevelopers to test the programme prototype. They each attended 10 training sessions, after which further programme adjustments were made based on their feedback.^22^ The resulting programme comprises eight steps: (1) identify service user’s primary and secondary goals; (2) identify and rank difficult situations and tasks; (3) introduce social skills training techniques and strengthen thought awareness; (4) introduce low-demand VR scenarios; (5) introduce simple VR social skills training scenarios; (6) introduce advanced VR social skills training scenarios; (7) social skills training in everyday situations outside VR; and (8) 3-month follow-up.^22^

### Inclusion criteria for the pilot study

The inclusion criteria included mental illness, long-term social isolation, social anxiety, and an interest in attempting to use a novel method under development. The criteria for exclusion included heavy substance use during the past 2 months and current suicidal ideation.

### Data collection

During the pilot period from November 2021 to May 2024, the mental health professionals collected data from the participants. They registered the following information about all participants: gender, age, previous treatment, background situation, personal goals, participant’s level of VR familiarity, number of consultations, and feedback from the individual user about their experience with the various elements of the programme. They also scored the participants according to the following instruments: Patient Health Questionnaire — 9 (PHQ-9), Generalized Anxiety Disorder (GAD-7) questionnaire, Work and Social Functioning (WSAS), and the Overall Anxiety Severity and Impairment Scale (OASIS). PHQ-9 is a widely used, rapid and effective tool for detection as well as for monitoring the severity of depression.^23^ The GAD-7 questionnaire is a seven-item, self-report anxiety questionnaire designed to assess the patient’s health status during the previous 2 weeks.^24^ WSAS is a simple 5-item reliable measure for impairment in functioning including mental health difficulties on their ability to function in terms of work, home management, social leisure, private leisure and personal or family relationships.^25^ OASIS is also a 5-item measure which can be used across anxiety disorders, with multiple anxiety disorders, and with sub-threshold anxiety symptoms.^26^

Also during the pilot period, health services researchers interviewed mental health professionals on several occasions to follow the progress. Meetings were held both physically and in VR (Horizon Workrooms). The interviews were recorded and transcribed using the Whisper’s language AI models.^27^

### Implementation

Practical courses were developed by the health services researchers to introduce mental health professionals to VR, and 35 employees attended these courses. The municipal mental health service established an internal VR group of employees, and the piloting of the programme was done by this group. Two mental health professionals delivered the programme to each of the 14 participants, and eight employees were involved in total. Employee turnover has caused the internal VR group to change a few times.

### Technological considerations

Two of the original project group members had technological competence in VR, and they shared experiences and knowledge with the rest of the group during the first phase of the project.

In the beginning, we used Oculus Quest and from May 2022 we also used Oculus Quest 2. In August 2022 Meta bought Oculus and changed the name to Meta Quest 2 and at the same time, they removed the restriction on personal user requirements so that the employees did not have to use personal information to log on. This topic was much discussed in one of the interviews, and as one of the healthcare professionals said, “We cannot require people [employees)] to log in with their personal Facebook accounts”. When they received the Meta Quest 2 headset, they had not expected this challenge to arise, but as they said, “Luckily for us, many others had also reacted to it”. This suggests that the meta-technology is mainly directed towards individual users rather than public services.

## Results

### Demographics and completion

A total of 14 service users were recruited to participate in the pilot of the programme. Seven participants completed the programme, two of these were female and five were male, while seven did not complete, two female and five male participants, see Table 1. A total of 102 consultations were carried out, ranging from 2 to 11 per participants.

**Table 1.** Demographics and completion of programme.

Participant number (p.nr.)	Gender	Age	Number of cons.	Completed
1	Male	20–24	11	Yes
2	Female	35–39	7	Yes
3	Male	20–24	5	No
4	Male	20–24	10	Yes
5	Male	20–24	12	Yes
6	Female	20–24	5	No
7	Male	25–29	11	Yes
8	Female	25–29	7	Yes
9	Male	40–45	10	Yes
10	Female	40–45	5	No
11	Male	40–45	2	No
12	Male	20–24	2	No
13	Male	18–19	10	No
14	Male	20–24	5	No

We include all 14 participants in the description to obtain a deeper understanding of the challenges faced by municipality mental health service users.

### Background history

The participants have similar personal histories. Common for all of them was that they lived socially isolated lives at the time they started on the social skills training programme. All had a certain degree of social anxiety, however various social and personal problems contributed to the complexity of their situation. Some lived alone and others lived with their parents. Participant 1: Somatic illness contributes to isolation to a certain extent. Low self-esteem. Work from home a few hours a week and receive disability benefits for hours not working. Participant 2: History of substance abuse and traumatic experiences. Has socially isolated herself for periods. Started job training just before entering the programme. Participant 3: Struggled with social phobia growing up. Are more capable today than before. Goes to the store, and orders a pizza. But rarely meets others outside the family. Struggles to make contact with new people. Interest in gaming, contact with others mainly via online games. Participant 4: Suffers from social anxiety and isolates himself in the boys’ room. Rarely outdoors. Only interacts with his mother and father. Has a few friends through online gaming. No other activities. Currently, on sick leave. Avoids difficult situations and becomes insecure about himself. Participant 5: Diagnosed with Asperger’s syndrome and depression. Has previously been employed. No meaningful activity in the form of work/school when entering the programme. Several suicide attempts in the past, the last one was one and a half years ago. Participant 6: Diagnosed with social anxiety and unspecified developmental disorder. Not completed upper secondary school, does not work. Inactive and very isolated. One online friend. Lives at home with her parents. Participant 7: Lives alone, and has previously had a supported job. Not completed upper secondary school. Bullied at school. Spends a lot of time and energy thinking about what others think of him. Participant 8: Autism diagnosis, currently studying, lives alone, experiences social anxiety. She also experiences a constant tiredness. Participant 9: Lives alone, has a supported job, limited contact with others. Has completed an education. Avoidant personality disorder. Struggled with social anxiety since childhood, experiences the symptoms as very life-limiting, a lot of avoidance and depressive symptoms, worsened in the last 6 months before starting the programme. Participant 10: Has experienced trauma in the past, and has little network. Lives with children. Growing up with psychological violence, lived with violent ex. Participant 11: Been more than 10 years in treatment in specialist mental health services. Has isolated himself, and has eating problems, PTSD, and anxiety disorders. Participant 12: No information was provided. Participant 13: Did not complete upper secondary school, in parallel with attending the programme he was examined for a somatic condition that affected his cognitive functioning, a history of being bullied, was suffering from depression when entering the programme. Participant 14: Socially isolated after completing upper secondary school. Had his suspicion of ADHD during the testing of the programme and was later diagnosed accordingly.

### Personal goals

The first step in the eight steps programme is to identify the service user’s personal goals and from the descriptions of goals given in Table 2, it is evident that they all strive for some normality in their life, and that it is not normal for them not to be socially active.

**Table 2.** Personal goals of the participants.

p.nr.	Personal goal	Goal achievement
1	To become safer in social settings, speak his mind more often, and be clearer to others about his boundaries.	Partially
2	To live a normal life and be able to take a bus, ask strangers for help, go to meetings and express her own opinions, and get a job.	Largely
3	To break out of social isolation and become more social/meet more people. To feel less anxious in social situations and experience more self-confidence. To challenge himself. Manage to ignore the anxiety more often.	No
4	To become less anxious and more confident in himself in conversation with others; know what to say, become better at small talk, overthink less, not care so much about what others think, to not push others away.	Largely
5	To practice social skills, to reduce discomfort in social situations. Become less uncomfortable with strangers and be better at conversations in general.	Largely
6	To get a job and participate in various activities.	Largely
7	To not care so much about what other people think about him.	Partially
8	To be able to take initiative to social gatherings with friends at school, to take the floor in a study context.	Partially
9	Get out more and meet others, difficult to specify more.	Partially
10	To participate in activities and get out of the house more often.	No
11	Wants social settings to be less demanding. In the long term, participate in the physical training clinic and contribute more as an uncle.	No
12	To reduce the social anxiety.	Partially
13	n.a.	No
14	To reconnect with old friends.	Largely

### Difficult situations

The second step of the program is to identify and rank difficult situations and tasks. The described situations were closely linked to their personal goals of what they wanted to achieve by participating in the social skills training programme. Communicating with strangers, small-talk, flirting, trying new activities and being better in social settings in general, came up in step two. Some have problems taking a bus, while others have problems entering a bar or a café.

### Observed effect for each of the participants

The two colleagues responsible for delivering the programme to participant 1 concluded that: “The goals have been partially achieved, and the participant experienced that he was better at taking the initiative in social settings than before he tested the programme, he was also more active and stood up for himself in a better way than before.”

For participant 2 they concluded “The sub-goals are considered to have been largely achieved. The participant felt that the timing of the VR programme and simultaneous work practice was very favourable.” Participant 3: “Stopped showing up to appointments and lost motivation on the way. He probably needed more than we could provide.” Participant 4: “We observe that he has made great progress since the start. Appears to be safer, takes up more space, dares to challenge, and seems happier than when we first met him.” Participant 5: “The participant experienced increased self-confidence in social settings. He has also become more aware of how he appears in social contexts, according to feedback throughout the programme. He considers that the VR programme was helpful.” Participant 6: “She didn’t complete all the steps because she felt ready to handle exposure in real life. Participant has an increased level of functioning considering that she is working and participating in activities. However, she feels that her anxiety has increased, which is natural when she participates more in social situations. She describes that she is in a better place now. She is much more out of her comfort zone and trying new things now. She describes that it is manageable, but that she still struggles with anxiety. She does more now, but is more tired. She is very happy that she is now at work and doing activities. She has, among other things, gone out to eat, been on a trip to a cabin, and went on a boat trip with colleagues.” Participant 7: “The goals are considered to have been partially achieved. The participant experienced at the end that he was better at taking initiative in social settings, was more active, and stood up for himself and communicated his boundaries better than before.” Participant 8: “At the last consultation, the participant considered that her social life has improved, however, she does not know if VR exposure is the reason. She recognises some benefits from VR. Actively used VR between the first 4 consultations, then more rarely. Exposure in vTime XR two times with employees from the mental health service.” Participant 9: “Reported positive experience and progress from using VR, however, it still seemed to be as difficult to expose himself in reality as before. The transfer value might be limited in his situation. The VR treatment has not led him to break his social isolation. He experienced some discomfort during the exercises and some progress was observed as he answered questions faster, with a higher volume in his voice.” Participant 10: “Completed five consultations and then wanted to end the training because she was unable to carry out the exercises because of her high level of anxiety.” Participant 11: “He was very interested in trying this treatment and completed two sessions. However, he did not want to continue the training as he had another service offer that he believed would help him more.” Participant 12: “He had only two consultations in the social skills training programme, and then wanted to join an anxiety group therapy and is now feeling better.” Participant 13: “The participant experienced gradual deterioration in functioning and increased levels of suicidal ideation. In agreement with the specialist mental health service, the contact with the municipality was ended. Very little focus on VR due to deteriorating health.” Participant 14: “He experienced a rapid improvement in functioning and did not feel that he needed more consultations. He had a job, and had made contact with old friends.”

### Feedback from the participants

One participant did not get triggered by the VR scenarios (participant 1), while others expressed that they liked the included scenarios and found some of them very relevant and challenging (participant 2, 3, 4, 5, 6, 7, and 8). Participant 2 said that VR was useful because it allowed her to train in a safe environment and that it was easy to achieve volume training.

Participants 8 and 9 found it especially useful to practice small talk in vTime XR, while participants 1,2 and 7 didn’t talk to strangers in vTime XR. Participant 1 found the VR chat function and found it to be an effective arena to practice small talk.

Participants 2, 3, and 9 would like the scenarios to have more variety, while most of the participants found the height of themselves in one of the scenarios to be wrong, while participant 4 found vTime XR to be too artificial to be experienced as real.

Participant 5 found the VR mask to have little space for his glasses, while his overall feedback was that VR has greatly helped him to break his social isolation.

Participant 2, 5, and 7 experienced that the scenarios were more effective in the beginning, but to a lower degree after some time. Participant 7 also thought that the questions popping up in the scenarios came and disappeared too quickly, and participant 8 shared this view.

Participant 6 thought it was cool to participate in the VR project, and she thinks it’s nice that we have such a modern concept in the municipality. She thinks that VR can be used to help people, even if some scenarios are of poor quality. Participant 4 shared her enthusiasm for VR.

### Experiences from the mental health professionals

Looking back at the 2,5 years-long process of recruiting and treating pilot users, some important insights came to the surface when the mental health professionals reflected and discussed in retrospect. One important experience seems to be that it was beneficial for pilot users to have an arena where they could practice what they learned from the VR programme in real life, such as sports, internship, job training or a social anxiety group. “They need a space to practice outside of VR, which made me reflect on the importance of these arenas”, said one of the professionals.

Some of the pilot users were on the autism spectrum or suspected to be. They adjusted the steps in the programme, for example, they practised small talk without the pressure of anxiety exposure in real life, which was essential for these pilot users. However, personal adjustments were also needed for some of the other pilot users, reflecting the complexity of their life situations and the need for flexibility.

Another insight is that the professionals find it hard to say who the VR training does not work for, as they have no information about the long-run impact. One participant was mentioned as an example where they did not observe any progress during the training period. However, they could not rule out possible progress in the longer run, as the participant was far from an activity where they could practice in real life (lack of arena). They could also see that for some participants it could be challenging to make use of the VR training when other major life challenges were going on at the same time. Another reflection was about the unpredictability of the usefulness for users, as “the initial VR training could be a small step along the way leading to something bigger”, as one of the professionals put it.

The VR technology itself was described as unique and very realistic, providing controlled environments for exposure therapy. In an interview with the internal VR group, they described how VR headsets like Quest 2 contributed to a more immersive experience, which is crucial for users to feel a sense of presence. They particularly highlighted how the new feature, of using hands in VR without controllers, offers a more realistic experience and enhances interaction. ”It is very fascinating that it gets more and more realistic, with the body language, and the use of the hands to have a more realistic communication”, one of them said.

As the pilot started just a few months before the global COVID-19 pandemic, there were several periods with a high degree of uncertainty. The mental health professionals describe how they were ready to get started with the piloting several times, but repeatedly had to stop due to the tightening of the local infection control regulations. One of them describes this experience as follows: “It was difficult to deal with. Can we start more sessions [with pilot users]? We were supposed to present the project to colleagues, but we couldn’t sanitise the VR sets between each person effectively. It is funny to think about now.”

To recruit service users for the pilot, the internal VR group had presentations at meetings with management and colleagues, and lunch breaks were used for hands-on testing of the VR sets. Managers and colleagues have shown engagement and interest throughout the project period.

When discussing why the recruitment was slow at the beginning of the project, one of them said: “In my experience, it’s hard to recommend something I’m not familiar with” — pointing at how it was up to each employee who acts as a contact person for a group of service users, to remember to suggest participation in the VR project and to promote it. Most of the pilot participants were recruited from the Youth Team, where three of the members of the internal VR group worked. They were strong advocates for the programme, however, a few participants were recruited from other parts of the service.

The professionals in this pilot study also found that they have become less reliant on pre-produced VR content throughout the project period. Their experience with vTime XR showed that each service user could have a unique and personalised experience based on their individual needs. Initially, we focused on finding and producing VR-content and identifying adaptable YouTube videos, but with vTime XR, the emphasis was more on how each user could benefit from being present in the immersive environment.

During this project, the mental health professionals have also found new ways to use the VR equipment. One of the employees, who was central in delivering the VR programme to the 14 users, was the contact person for one service user with autism who they experienced problems finding a position where they could assist. The patient was isolated at home with his parents, and different strategies had been tried without success, and there was an increasing concern for the physical and mental health of the patient. The contact person got the idea to try VR and the patient and the mother borrowed VR sets from the project, and the patient was able to meet the therapists in a VR room where the mother also attended. They played in various apps, and through this, they built a relationship with the patient. They received positive feedback from the patient, parents, and allied services because they found a creative solution using VR to connect with the patient. This show that when mental health professionals started to explore the technology, new areas of potential use emerged as they became more familiar with the technology and gained more experience.

## Discussion

The results from this pilot study show that even though many of the service users have some of the same experiences in life, and they all want to become more socially active, there is great variation in how they experience social skills training in VR. Some experienced a limited effect, while others experienced a substantial effect and managed to enter the step where they practised social skills in real life. Some just needed some tips on what to do in situations where they have to talk to strangers, while others needed a long time of training before they were ready to talk to people they didn’t know.

Mental health professionals assess the individual service users’ needs and suggest a plan or method they have the most faith in. Such important assessments are made every day for many of the mental health service users, however now, this municipality has a new tool that they can utilise, if deemed appropriate.

Although there were comments about the quality of the pre-produced VR scenarios, like the height of the ”I person”, the majority of the participants experienced various forms of immersive presence. When learning skills, this characteristic of the VR technology, that users are able to ”trick the brain” in order to be present in the scenario, can be beneficial. A recent RCT study that included 134 university students found that the sense of presence was higher in immersive versions of a skill training programme than in a desktop version.^28^ However, even VR training programs for developing social skills are found to perform better than alternative training programs, much is still unknown about the effect of the content of these programs and the specific contribution of the VR technology.^29^

### Barriers still present

During this almost six-year-long project that includes this pilot study, we have learned that there still are barriers to the implementation of VR technology in municipal mental health services. These are first and foremost related to technical maintenance of the equipment, especially troublesome with person-centred users logging into the VR sets. And software updates that are typically required when the set should be ready for use.

There are also barriers related to the extent of high-quality scenarios available, relevant, and adequate for mental health service users. Other researchers have also concluded that the primary limiting factor for the implementation of VR in clinical practice currently is the lack of evidence-based VR programs that can be purchased off the shelf and used by clinicians.^16^

The staff competency and confidence is previously found to be a barrier when implementing VR exposure therapy in health care services,^18^ however this was not a barrier identified in this pilot study, suggesting that the developed practical course completed by 35 employees and managers gave them sufficient competence and confidence.

Previous research emphasises that successful implementation in a healthcare setting is dependent on health professional and service readiness for change, leadership at the local service level, the appropriateness, and responsiveness of the technology for the target end users, and, critically, funding models being available to support implementation.^30^

Through the development of the 8-step social skills training programme and the pilot study, the researchers have followed the efforts and development among mental health professionals in the municipality. The employees have been given freedom by the management to explore VR, and they have established an internal project group of mental health employees who believe that VR technology can be of help to service users.

Staff turnover created a certain instability during the pilot study that made continuity in exploration of VR somewhat demanding. However, the effort from the internal project group to inform and engage their colleagues, contributed to other employees being recruited and trained if someone changed jobs, ensuring continuity in the project.

Other municipalities have made contact to hear about their experiences with VR. External funding was necessary for conducting this research, as municipal budgets do not necessarily provide good conditions for carrying out research. However, we stress that it is important that all municipalities that explore new types of treatment collaborate with researchers who can document what is being done, so that other municipalities that want to explore new approaches to solving mental and social problems faced by the population living in the municipality, have a knowledge base to build on to develop the best possible methods and solutions in the long run.

In this project, we observed that the mental health professionals involved had high readiness for exploring VR technology, the management was supportive, and the service users were willing to try the developed programme. This supports the findings of previous research about integrating digital health solutions into mental health service.^30^

### Strengths and limitations

We did not set out to identify an effect of the social skills training program, rather, we wanted to explore how a program that involved VR technology could be implemented in a municipal mental health service setting. Still, a limitation of the study includes the small sample size.

### Further research

The collaborative research group does not recommend proceeding with a large-scale time- and cost-consuming randomised controlled trial to identify the effect of the programme that has been developed. Although municipal mental health services must provide evidence-based treatment to the greatest extent possible, individual variation suggests that it is difficult to predict the effect for each service user before trying. An RCT study could show that the program had an effect on social isolation on average for a large group of mental health service users, however, they still would not know in advance who would benefit from the VR training.

To our knowledge, this is the first social skills training study using VR in primary mental health services. The development of relevant, flexible, and high-quality VR scenarios should be prioritised by the mental health authorities and municipalities, as it has the potential to help socially isolated persons become more socially active and thus increase their quality of life.

Because many municipalities are small and have few mental health professionals, larger municipalities should take responsibility for the development of VR-scenarios of high quality that also could be used in smaller municipalities - if research confirm that these are effective tools in treatment of social or mental health problems.

## Conclusion

The results from this pilot study suggest that social skills training in VR could have a positive effect on service users in a municipal mental health setting. Some experienced a limited effect, while others experienced a substantial effect. The participants had different levels of readiness for training in real life. Some just needed some tips on what to do in situations when meeting strangers, while others needed a long time of training and support before they were ready to talk to people they did not know. In particular, we suggest that social VR as VTime, can provide a new and safe environment for mental health service users for practising social skills, with close support from the mental health professionals.

VR technology is now available as a new tool for mental health professionals in the service this pilot study was conducted. Thus, VR is implemented as a new element in social skills training in the third-largest municipality in Norway. However, how they utilise the technology in the future, will depend on the incorporation of VR in the ongoing continuous development of mental health services.
